# Root canal dressings for revascularization influence *in vitro* mineralization of apical papilla cells

**DOI:** 10.1590/1678-7757-2018-0396

**Published:** 2019-04-11

**Authors:** Juliana Garuba RAHHAL, Emanuel da Silva ROVAI, Marinella HOLZHAUSEN, Celso Luiz CALDEIRA, Carlos Ferreira dos SANTOS, Carla Renata SIPERT

**Affiliations:** 1 Universidade de São Paulo, Faculdade de Odontologia, Departamento de Dentística, São Paulo, São Paulo, Brasil.; 2 Universidade de São Paulo, Faculdade de Odontologia, Departamento de Estomatologia, São Paulo, São Paulo, Brasil.; 3 Universidade de São Paulo, Faculdade de Odontologia de Bauru, Departamento de Ciências Biológicas, Bauru, São Paulo, Brasil.

**Keywords:** Anti-bacterial agents, Calcium hydroxide, Cultured cells, Endodontics

## Abstract

Endodontic revascularization is based on cell recruitment into the necrotic root canal of immature teeth after chemical disinfection. The clinical outcome depends on the ability of surviving cells from the apical tissue to differentiate and promote hard tissue deposition inside the dentinal walls.

**Objective:**

To investigate the effect of calcium hydroxide (CH) and modified triple antibiotic paste (mTAP – ciprofloxacin, metronidazole and cefaclor) on the viability and mineralization potential of apical papilla cells (APC) *in vitro* .

**Material and Methods:**

APC cultures were kept in contact with CH or mTAP (250-1000 µg/mL) for 5 days, after which cell viability was assessed using 3-(4,5-Dimethylthiazol-2-yl)-2,5-diphenyltetrazolium bromide (MTT) assay. Next, APCs were subjected to CH or mTAP at 250 µg/mL for 5 days before inducing the differentiation assay. After 14 and 21 days, calcium deposition was assessed by the Alizarin Red S staining method, followed by elution and quantification using spectrophotometry. Data were analyzed using ANOVA followed by Tukey *post hoc* test.

**Results:**

CH induced cell proliferation, whereas mTAP showed significant cytotoxicity at all concentrations tested. APC treated with CH demonstrated improved mineralization capacity at 14 days, while, for mTAP, significant reduction on the mineralization rate was observed for both experimental periods (14 and 21 days).

**Conclusion:**

Our findings showed that CH induces cell proliferation and improves early mineralization, whereas mTAP was found cytotoxic and reduced the mineralization potential *in vitro* of APCs.

## Introduction

Endodontics is the branch of dentistry that deals with the treatment of diseases affecting the dental pulp and apical tissues. The treatment of permanent immature teeth with necrotic pulp remains as one of the most important challenges for contemporary Endodontics ^1^ . For many decades, dentists have used calcium hydroxide to aid in the formation of a calcified barrier in the apical region of an immature root ^2^ . With this purpose, frequent replacement of medication is necessary, thus prolonging the time of treatment completion and leading to increased probability of root fracture and tooth loss ^3^ . The use of an artificial apical barrier with Mineral Trioxide Aggregate (MTA) is a better alternative to conventional apexification, since it has the distinct advantage of being a one or two-step treatment. Although both procedures are widespread in their use, they result in thin-walled, short roots, leading to a significant structural impairment of the tooth ^4^ ^,^ ^5^ .

Currently, revascularization has been proposed as a new treatment protocol for immature teeth with pulpal necrosis ^6^ . In this context, a blood clot is induced into the pulpal cavity after root canal disinfection with intracanal antimicrobials. This treatment is known for leading to an increase in root thickness and eventually in length as well, resulting in a better prognosis for the structural outcome of these teeth ^4^ ^,^ ^6^ . The main mechanism involved in these procedures is suggested as the migration of mesenchymal cells from apex surrounding tissues into the canal lumen, giving rise to the mineralized tissue formation ^7^ . Nevertheless, the success of this kind of therapy depends simultaneously on the maximum disinfection of pulpal cavity and host cells function maintenance ^8^ .

Since root canal walls are not mechanically cleaned during revascularization treatment, irrigation and intracanal dressings have a pivotal role in pulpal cavity disinfection. The induced blood clot then brings the apical tissue cells into the disinfected pulpal cavity, which, in turn, differentiates and results in hard tissue deposition inside the dentinal walls ^7^ . Due to its high pH, calcium hydroxide was initially discouraged as an intracanal dressing for revascularization treatment ^9^ , and triple antibiotic paste was proposed as an interappointment dressing instead ^6^ . However, *in vitro* studies have demonstrated higher biocompatibility of calcium hydroxide compared to other antibiotic combinations in both direct ^10^ and indirect cytotoxicity ^11^ ^,^ ^12^ . On the other hand, in a clinical study comparing the triple antibiotic paste with calcium hydroxide, Bose, et al. ^13^ (2009) found higher increase in dentin wall thickness for TAP when compared to CH. These data suggest the need to understand not only cytotoxicity, but also the late effect of the medications on the mineralization potential of APCs.

TAP ^14^ was proposed as the first choice for intracanal dressing at revascularization protocols ^6^ . However, cytotoxicity studies pointed to a significant toxic effect of antimicrobials on APC *in vitro* ^10^ ^,^ ^15^ , specially due to minocycline ^16^ , which, in turn, is also responsible for the discoloration caused by TAP ^17^ . Additionally, TAP has already been observed to negatively modulate SCAP differentiation *in vitro* , even at low concentrations ^15^ . Considering these issues together, the proposed formula, which replaces minocycline with cefaclor ^10^ ^,^ ^18^ , was used in this study (modified TAP – mTAP). Despite evidence ^15^ already showing reduced differentiation rates in TAP-treated APC, the effect of mTAP compared to CH under this context remains still undetermined.

Most studies in this field have investigated the cytotoxicity of substances based on cell viability without considering the functional state of the cells that have survived the intracanal dressing process. Therefore, the aim of this study was to investigate the late effect of calcium hydroxide and modified triple antibiotic paste on the differentiation potential of apical papilla cells (APCs) *in vitro* .

## Material and methods

### Culture of human APC

All experiments were conducted in accordance with the Declaration of Helsinki. Ethical approval was obtained from the Ethics Committee of Human Research from the Institution (CAAE 45748615.4.0000.0075). Third molars (n=3) with incomplete root formation were obtained with the patients’ informed consent. The apical papilla was manually separated from the roots. Tissues were minced and incubated for cell growth in Minimum Essential Medium Eagle – Alpha Modification (α-MEM) (Invitrogen, Thermo Fisher, Waltham, Massachusetts, USA) with 15% fetal bovine serum (FBS) (Gibco, Thermo Fisher, Waltham, Massachusetts, USA), glutamine (2 mM – Invitrogen, Thermofisher, Waltham, Massachusetts, USA), L-ascorbic acid phosphate (0.1 mM – Sigma-Aldrich, St. Louis, MO, USA), and antibiotics (100 µg/mL penicillin, 100 µg/mL streptomycin, 0.5 mg/mL amphotericin B – Invitrogen, Thermofisher, Waltham, Massachusetts, USA) (Proliferation Medium – PM) under standard culture conditions of 37ºC, 100% humidity, 5% CO_2_, and 95% air. The cells were used between the second and fourth passages ^19^ ^,^ ^20^ .

### Phenotypic characterization of APC

The cultured cells were characterized based on their mesenchymal origin by immunostaining for vimentin. Once fixed in acetone, they were stained with mouse anti-vimentin (Santa Cruz Biotechnology, Dallas, TX, USA) followed by DyLight 594 conjugated anti-mouse IgG (Vector Labs, Burlingame, CA, USA). The slides were mounted with a mounting medium containing DAPI (4’,6-diamidino-2-phenylindole dihydrochloride hydrate) to stain the nucleus. The images of these cells were captured by a fluorescence microscope (Leica TCS-SPE; Leica, Wetzlar, Hesse, Germany) ^21^ .

### Preparation of antimicrobials

Calcium hydroxide (CH) (Biodinâmica Química e Farmacêutica LTDA, Ibiporã, PR, Brazil) and modified Triple Antibiotic Paste [mTAP – ciprofloxacin, metronidazole, and cephalosporin (1:1:1)] (Farmácia Fórmula & Ação, São Paulo, SP, Brazil) were tested. Stock solutions were prepared using “ready to use” powdered medications reconstituted in α–MEM at 5 mg/mL. The solutions were sterilized prior to use by filtering them with 0.22 µm pore size microfilters (Millipore, Billerica, MA, USA) and serially diluted to the final concentrations (1000, 500 and 250 µg/mL) ^10^ ^,^ ^11^ .

### Cell viability

APCs were seeded on a 96-wells plate at 8 × 10^3^ cells/well. The medium was replaced after 24 h with 100 µL of plain culture medium or containing diluted intracanal dressings in triplicate. Decreasing concentrations of CH or mTAP solutions were tested at 1000, 500, and 250 µg/mL. Cytotoxicity was determined after 5 days by using MTT [3-(4,5-Dimethylthiazol-2-yl)-2,5-diphenyltetrazolium bromide] (Sigma-Aldrich, St. Louis, MO, USA) in phosphate buffered saline (Merck, Kenilworth, Nova Jersey, EUA) (5 mg/mL – 20 µL), followed by 180 μL of α-MEM 15% FBS. After 4 h, the solutions were discarded and replaced by 100 µL of dimethyl sulfoxide (Sigma-Aldrich, St. Louis, MO, USA). Optical density was determined using a plate reader (Synergy HT, Biotek, Instruments, Inc. Winooski, VT, USA) at the wavelength of 570 nm ^10^ ^,^ ^22^ . Quantitative analysis (%) was performed by normalizing the data with the ones from the untreated control group (medium only).

### Mineralization assay

The mineralization assay was performed in triplicate using a 48-wells plate (8×10^3^ cells/well). APCs were kept in contact with CH or mTAP at 250 µg/mL in α–MEM 15% FBS solution or in plain culture medium for 5 days. The culture medium was discarded and replaced with a differentiation medium (DM): PM added by 0.01 µM dexamethasone and 1.8 mM monopotassium phosphate ^20^ . After 14 and 21 days, the cells were subjected to 2% Alizarin Red S staining (Sigma-Aldrich, St. Louis, MO, USA) to detect calcium deposition. For quantification, the recovery was performed by elution with 200 µL of 10% ammonia hydroxide, followed by absorbance measurement at 405 nm (Synergy HT, Biotek, Instruments, Inc. Winooski, VT, USA) ^23^ . Quantitative analysis (%) was performed by normalizing the absorbance data from samples with the ones of the positive control group (untreated DM).

### Statistical analysis

Statistical analysis was performed using the Graph Pad Prism 5.0 software (GraphPad Software, La Jolla, USA). Data were subjected to one-way analysis of variance followed by Tukey’s post-test for comparison between the pairs of the groups, with a significance level of p<0.05.

## Results

**Figure 1:**
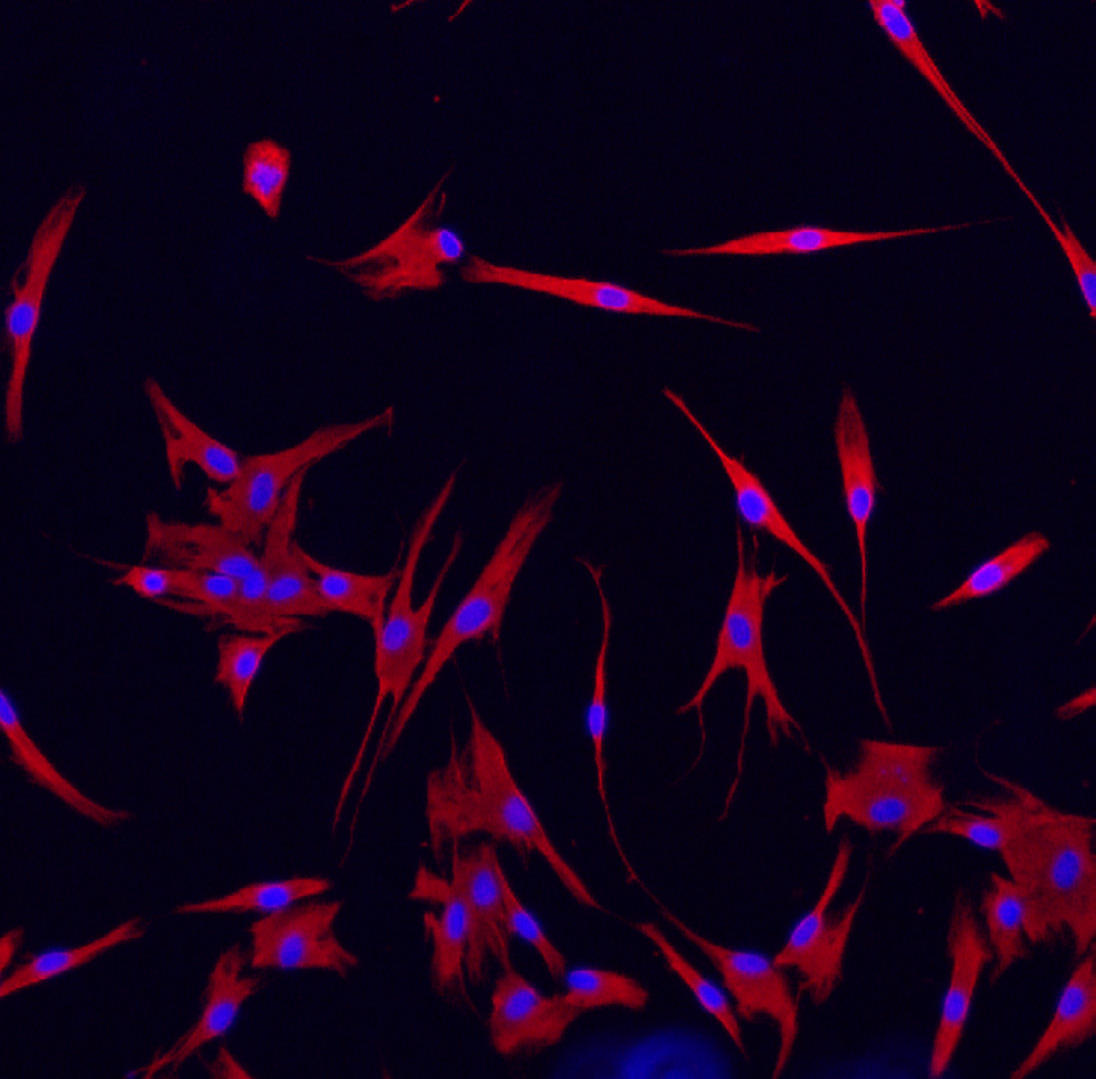
Characterization of human apical papilla cell (APC) culture by vimentin. Cultured APCs were immunostained for vimentin (red). Image captured by a fluorescence microscope at 40X

Cell culture and phenotypic characterization of APC are shown in Figure 1 . The mesenchymal origin of the cells was demonstrated by vimentin immunostaining, and fibroblast-like morphology is evidenced in the cultured cells.

### Cell viability

**Figure 2:**
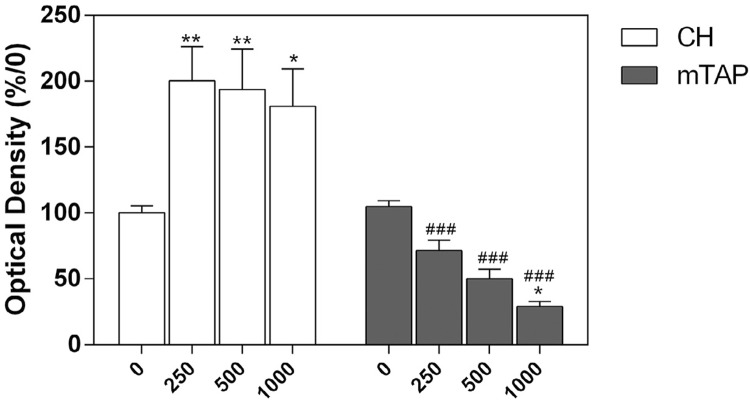
CH and mTAP cytotoxicity on APCs at the indicated concentrations for 5 days. Cell viability detected by MTT assay and normalized based on control (untreated) cells. *P<.05 and ***P<.001 in comparison to culture medium alone (0) (n=9

Cell viability data are shown in Figure 2 . Once in contact with CH, cellular metabolic activity was upregulated at all dilutions of CH, suggesting cellular proliferation. On the other hand, mTAP significantly reduced cell viability at all concentrations tested. The cells kept in contact with 250 µg/mL of mTAP showed a survival rate of 71.56%. Hence, this concentration was selected for the mineralization assay.

### Mineralization assay

**Figure 3:**
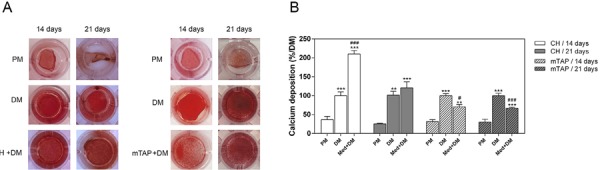
Mineralization potential of APC assessed through calcium deposition by using Alizarin Red S staining. Once kept in contact with CH or mTAP at 250 µg/mL for 5 days, osteogenic mineralization was induced for 14 and 21 days. A – macroscopic view of wells after staining; and B – quantification of calcium eluates normalized by positive control (DM). PM: proliferation medium, DM: differentiation medium; CH + DM and mTAP + DM: calcium hydroxide or mTAP prior to mineralization; Med + DM: medication prior to mineralization. **P<0.01 and ***P<0.001 in comparison to PM; #P<0.05 and ###P<0.001 in comparison to DM (n=9)

The mineralization potential of APCs was assessed after 14 and 21 days using Alizarin Red S staining ( Figure 3 ). The cells kept in contact with CH prior to differentiation induction (CH+DM) demonstrated stronger calcium deposition after 14 days in comparison to basal differentiation cells (DM). After 21 days, mineralization of CH-treated cells (CH+DM) was similar to the differentiation control (DM). Nevertheless, the cells treated with mTAP (mTAP+DM) demonstrated a significant decrease in calcium deposition in comparison to the differentiation control (DM) in both experimental periods (14 and 21 days – Figure 3B ).

## Discussion

The treatment of immature teeth with necrotic pulp is one of the most challenging tasks in contemporary Endodontics. Regeneration protocols offer the possibility of tooth structure reinforcement due to thicker and eventually longer root canal walls compared to conventional apexification procedures ^6^ . Given this context, the requirements for successful revascularization in Endodontics should comprise the maximum disinfection of the pulpal cavity and the minimal harmful effect to the host cells ^8^ . Clinically, stem cells from apical papilla are not disjointed from other mesenchymal cells from the whole tissue, so keeping this heterogenic environment *in vitro* might be important when investigating the cellular behavior under contact with chemical substances. Therefore, the biological condition of the cells that survive after antimicrobial disinfection of the canals should be considered for the success of revascularization treatment. Therefore, it seems reasonable to understand how damaged apical cells might be after diffusion of antimicrobials used to treat immature teeth with necrotic pulps.

In order to avoid increased root wall fragility, mechanical removal of bacteria and necrotic tissue is not performed ^1^ ^,^ ^6^ . Thus, procedures of pulpal cavity disinfection become critical for revascularization. Microbiological control is achieved by using irrigating solutions and intracanal dressings. Initially, the protocol proposed by Banchs and Trope ^6^ (2004) defined triple antibiotic paste as an intracanal dressing. Some authors rejected the use of calcium hydroxide for revascularization procedures ^9^ due to its high pH, which, in turn, would result in tissue/cells necrosis. However, studies have demonstrated the superior cytotoxicity of TAP instead ^10^ ^,^ ^11^ ^,^ ^15^ . Under the same concentration (1 mg/mL), TAP (metronidazole, ciprofloxacin, and minocycline) and double antibiotic paste (DAP – metronidazole and ciprofloxacin) induced cell death, while calcium hydroxide increased cell proliferation ^10^ . At 7 days of contact, Phumpatrakom, et al. ^15^ (2014) observed complete cell death with 1 mg/mL of TAP and proliferation inhibition with 0.39 µg/mL. At 5 and 7 days of medication contact, Chuensombat, et al. ^11^ (2013) noticed significant toxicity for TAP, minocycline and ciprofloxacin at 25 µg/mL, but not for metronidazole. Minocycline, in turn, was the only antimicrobial resulting in less than 70% of viability at 6.25 µg/mL. In this study, mTAP used at 250 µg/mL, a concentration much closer to the one employed at the Endodontics practice, rendered a survival rate of above 70%, but it was still harmful enough to decrease the late mineralization potential of surviving APCs.

The abovementioned studies have already reported patterns of cytotoxicity and cell proliferation induced by intracanal antimicrobials. However, the substances used as intracanal dressing for necrotic pulps in teeth with open apices might easily diffuse into the surrounding living tissues through the large apical foramen. The study by Bose, et al. ^13^ (2009) showed improved increase in dentin wall thickness for calcium hydroxide when placed at the coronal half of the root canal. The increase in wall thickness was more than 50% superior than when CH was placed into the apical tissue. One may speculate that APCs might be better stimulated when submitted to low concentrations of CH during treatment. In this study, the concentration of 250 µg/mL was chosen for both medicaments considering the survival rate superior to 70% for mTAP [cut-off recommended by international standards for evaluation of medical materials (ISO 10993-1:2018)]. Based on the viability assay, calcium hydroxide induced cell proliferation and mTAP induced the lesser cytotoxic rate (28.44%) at this concentration, thus corroborating data from previous studies ^10^ . Our data showed that keeping the cells in a low concentration of calcium hydroxide was enough to improve *in vitro* mineralization in 14 days ( Figure 3 ). In 21 days, however, no difference was observed compared to the differentiation control. We suggest that the substance might improve early differentiation and accelerate the biological process of hard tissue deposition. On the other hand, cells kept in contact with mTAP prior to differentiation showed a lesser mineralization rate in comparison to the differentiation control ( Figure 3 ).

The improvement in cell mineralizaton by CH might be due to growth factor release modulation. On dental pulp cells *in vitro,* calcium containing materials increased Transforming Growth Factor (TGF)-β1 secretion ^24^ and upregulated the gene expression of osteopontin and bone morphogenetic protein-2 ^25^ . On SCAP, TGF-β1 was shown as a proliferative growth factor and alkaline phosphatase (ALP) activity inducer at low concentrations ^26^ . On murine dental pulp cells, CH was shown to induce cell differentiation through the gene expression of EphB2 ^27^ , which, in turn, controls the gene expression of alkaline phosphatase, osterix, and bone sialoprotein. However, determining the precise mechanisms involved with improved proliferation and mineralization is therefore an issue for further investigations.

Contrastingly, in periodontal ligament fibroblasts, TAP was observed to be more cytotoxic than CH, and it was also able to induce interleukin-6 mRNA in a pro-inflammatory context ^16^ . Pro-inflammatory cytokines were found responsible for decrease in both late cell proliferation and mineralization with decrease in calcium deposition and ALP activity ^28^ . In the subcutaneous tissue of mice, TAP implants induced a stronger inflammatory process compared to CH and empty tubes ^29^ , demonstrated by the increase in inflammatory infiltrate and mediators. The precise inflammatory mediators potentially induced by mTAP and their role on the deleterious effects raised by mTAP on apical papilla cells is an issue for future studies. In summary, when considered together, these data suggest that CH and antibiotics containing pastes might differentially modulate inflammatory mediators and growth factors that, in turn, would control the differentiation rate of APCs *in vitro.* The role of these substances on the milieu of growth factors released by this cell population is an issue that requires further investigations.

In conclusion, our study demonstrated that, while CH induces cellular proliferation and improves early *in vitro* mineralization, mTAP was found to be more cytotoxic and to decrease the differentiation potential of APCs *in vitro.*
